# Cardiac implantable electronic device malfunction after pulsed field ablation—insights from the MAUDE database

**DOI:** 10.1093/europace/euag092

**Published:** 2026-04-22

**Authors:** Tasuku Yamamoto, Jakub Sroubek, Justin Lee, Roy Chung, Koji Higuchi, Arwa Younis, Walid Saliba, Ayman Hussein, Oussama Wazni, Pasquale Santangeli

**Affiliations:** Electrophysiology and Pacing Section, Cleveland Clinic, 9500 Euclid Avenue, Cleveland, OH 44195, USA; Electrophysiology and Pacing Section, Cleveland Clinic, 9500 Euclid Avenue, Cleveland, OH 44195, USA; Electrophysiology and Pacing Section, Cleveland Clinic, 9500 Euclid Avenue, Cleveland, OH 44195, USA; Electrophysiology and Pacing Section, Cleveland Clinic, 9500 Euclid Avenue, Cleveland, OH 44195, USA; Electrophysiology and Pacing Section, Cleveland Clinic, 9500 Euclid Avenue, Cleveland, OH 44195, USA; Electrophysiology and Pacing Section, Cleveland Clinic, 9500 Euclid Avenue, Cleveland, OH 44195, USA; Electrophysiology and Pacing Section, Cleveland Clinic, 9500 Euclid Avenue, Cleveland, OH 44195, USA; Electrophysiology and Pacing Section, Cleveland Clinic, 9500 Euclid Avenue, Cleveland, OH 44195, USA; Electrophysiology and Pacing Section, Cleveland Clinic, 9500 Euclid Avenue, Cleveland, OH 44195, USA; Electrophysiology and Pacing Section, Cleveland Clinic, 9500 Euclid Avenue, Cleveland, OH 44195, USA

**Keywords:** Pulsed field ablation, Pacemakers, Defibrillators, Device malfunction, CIED

Pulsed field ablation (PFA) is rapidly replacing radiofrequency ablation in catheter-based treatments for cardiac arrhythmias, owing to improved safety profile and shortened procedural duration.^1^ Although it is generally well accepted that the risk of collateral tissue damage is smaller with this non-thermal energy source, less is known about its safety in patients with cardiac implantable electronic devices (CIEDs). Recent case reports describing CIED damage following PFA raised the alarm about the potential of such interactions.^2,3^ The magnitude of this problem is unclear. Furthermore, because multiple manufacturers offer different platforms and configurations for PFA, there may be a product- or manufacturer-specific patterns in adverse effects of PFA on CIEDs. In this study we systematically examined reported cases of adverse effects of PFA on CIED functions.

Manufacturer and User Facility Device Experience (MAUDE) Database was searched for CIED malfunctions following PFA, using the combination of commercially available PFA device product names (pulsed field ablation, Farapulse, Affera, Varipulse and PulseSelect) and CIED type (pacemaker or defibrillator) as keywords. We excluded reports on lead dislodgements or induction of arrhythmias due to PFA-CIED interactions, as the focus of the report was the CIED malfunctions after PFA. Duplicate reports of a single incident from different parties were identified through event descriptions and dates of occurrence. Because MAUDE is a publicly available anonymized database, the need for ethical approval was waived.

We identified 29 reports on CIED malfunctions related to PFA procedures. Six reports were excluded as duplicate submissions, leaving 23 individual events to be analyzed (*Table 1*). Overall, 19 out of 23 incidents (83%) involved an implantable cardioverter-defibrillator (ICDs) or cardiac resynchronization therapy defibrillators (CRT-Ds) across different manufacturers including Boston Scientific, Medtronic and St Jude Medical. In 13 out of 23 (57%), the PFA system was identified as the Affera system (Medtronic), and Farapulse system (Boston Scientifc) in three (13%). There were nine occurrences of CIED generator failure (three Boston Scientific, two Biotronik, one Medtronic, one St Jude Medical and two unknown manufacturer’s defibrillators; six with Medtronic Affera and one with Boston Scientific Farapulse system), eight reported cases of device lead-related complications (at least two atrial leads, four right ventricular defibrillator leads and two left ventricular leads; six with Medtronic Affera systems and two with Boston Scientific Farapulse system), two cases of loss of capture from leadless pacemakers (one Micra from Medtronic and one Aveir from St Jude Medical; PFA manufacturers unknown) and four cases of defibrillator reset into backup mode operation (all St Jude Medical devices; one Affera and three unknown PFA systems) (***Table 1***). No report specifically suggested the use of any non-approved or investigational system.

**Table 1:** CIED malfunction following PFA from the MAUDE database

Event type	CIED device (Manufacturer)	PFA device (Manufacturer)	Event description	PFA location	Consequences
Generator failure	Cobalt XT DR (Medtronic)	Unknown	ICD failed to pace	Unknown	Generator change
Generator failure	Dynagen X4 CRTD (Boston)	Affera	Unable to interrogate	Dislodged LV lead	Generator change
Generator failure	Resonate X4 CRT-D (Boston)	Affera	Unable to interrogate	SVC coil	Generator change
Generator failure	Dynagen EL ICD DR (Boston)	Unknown	Unable to interrogate	Unknown	Generator change
Generator failure	ICD (non-Medtronic)	Affera	Unable to interrogate	CTI near ICD lead	Generator change
Generator failure	ICD (non-Boston)	Farawave	Unable to interrogate	RSPV near SVC coil	Unknown
Generator failure	Ellipse DR (SJM)	Affera	↓ System impedance	LV, 3 cm from RV lead	Generator change
Generator failure	Ilivia 7 VR-T (Biotronik)	Affera	Device damage	LV septal apex	Generator change
Generator failure	Ilivia Neo 7HF-T (Biotronik)	Affera	Device damage	LV septal apex	Generator change
Lead-related	Pacemaker (unknown)	Farawave	↑ RA threshold (1.1 to 3.6 V)	RSPV near RA lead	Reprogramming
Lead-related	ICD (non-Boston)	Farawave	Noise ↑ RV threshold	PVI only	New lead addition
Lead-related	CRTD (non-Medtronic)	Affera	LV lead threshold rise	Unknown	Battery 7.5 > 4.5 years
Lead-related	CRTD (unknown)	Affera	↓ RV sensing ↑ LV threshold	Unknown	Reprogramming
Lead-related	ICD (non-Medtronic)	Affera	↑ RV lead threshold	Unknown	Reprogramming
Lead-related	CRTD (unknown)	Affera	↓ RV sensing ↑ RV thresholds	Unknown	↓ Battery longevity
Lead-related	CRTD (unknown)	Affera	RV lead exit block	Unknown	Unknown
Lead-related	Pacemaker (unknown)	Affera	Atrial lead non-capture	Anterior RIPV	Reprogramming
Leadless pacemaker	Micra (Medtronic)	Unknown	Loss of capture	Unknown	Reprogramming
Leadless pacemaker	Aveir (SJM)	Unknown	Loss of capture	Unknown	Aveir extraction TV-PPM
Program reset	Gallant HF (SJM)	Unknown	Reset to backup mode	PVI	Reprogramming
Program reset	Gallant HF (SJM)	Unknown	Reset to backup mode	Unknown	Reprogramming
Program reset	Gallant DR (SJM)	Unknown	Reset to backup mode	Unknown	Reprogramming
Program reset	Gallant DR (SJM)	Affera	Reset to backup mode	LV anterior wall	Reprogramming

Of the incidents reported so far, particularly concerning are the reports on permanent generator damage leading to system shutdown and device replacements, which seems to happen more often than in radiofrequency ablation.^4^ Current CIEDs are designed with protective circuits against high-voltage currents, safeguarding these devices from radiofrequency or direct current cardioversions. However, the very high-voltage generated by PFA may overwhelm those protective mechanisms, resulting in permanent damage to the pulse generators.^5^ Analyses on the generators in the aforementioned cases of permanent CIED failure following PFA suggested damage to the protective diodes and transistors.^2,3^ A previous report suggested that the application of PFA close to the defibrillator coil might have been responsible for CIED dysfunction,^2^ and five cases in the current series reported use of PFA in close proximity to the CIED leads. Theoretically maximizing the distance between PFA catheter and CIEDs may decrease the risk of such interactions. However, four cases in our series reported the use of PFA only in the left ventricle, suggesting that PFA at relatively remote locations from the leads may still result in significant CIED interaction. Furthermore, from a clinical perspective maintaining adequate distance from CIED components may not be always possible as some arrhythmia substrates may be in proximity to CIED components. These considerations may be of relevance especially with the expanding PFA use for ventricular arrhythmias,^6–8^ given the potential for PFA applications in proximity to ventricular leads, as well as the high prevalence of CIED in this patient population.

Another important observation is that CIEDs from any manufacturer can be vulnerable to such undesired interactions.^3^ With regard to PFA systems, Affera system was the most commonly reported device in our dataset, followed by Farapulse system, with no reports on PulseSelect or Varipulse systems. It is possible that variable catheter designs and electrical field strength may result in different risk profiles among platforms. However, caution should be exercised when interpreting these results, as they may simply reflect variable adoption rates of these catheters, as well as differing reporting patterns across individual manufacturers, and lack of MAUDE reports should not exclude the possibility that a specific device causes CIED malfunctions. Nevertheless, it may be worth noting that Affera system is the only monopolar PFA device currently approved by Food and Drug Administration (FDA). Given its increased electric field distribution and propensity to affect remote tissues,^9,10^ monopolar PFA may interact differently with CIEDs from bipolar PFA.

The present analysis should be viewed in the context of several limitations. First, as the precise number of CIED patients who had undergone PFA is unavailable, it is difficult to estimate the exact frequency of these events. It should also be emphasized that because of the voluntary nature of the MAUDE database, it is likely that the magnitude of this problem is vastly underestimated. Moreover, details including the peri-procedural device programming and precise PFA catheter locations and relationship with CIED leads locations were not available in the majority of cases. Nevertheless, the current report raises awareness on the potential scale and diversity of the problem, and we urge the operators to be aware of these risks and exercise caution when PFA is performed in patients with CIEDs. Device interrogations should be routinely performed before and after PFA.

## Data Availability

The data underlying this article will be shared on reasonable request to the corresponding author.
